# A Versatile Micromanipulation Apparatus for Biophysical Assays of the Cell Nucleus

**DOI:** 10.1007/s12195-022-00734-y

**Published:** 2022-09-06

**Authors:** Marilena L. Currey, Viswajit Kandula, Ronald Biggs, John F. Marko, Andrew D. Stephens

**Affiliations:** 1 Biology Department, University of Massachusetts Amherst, Amherst, USA; 2 Department of Molecular Biosciences and Department of Physics & Astronomy, Northwestern University, Evanston, USA; 3 Feinberg School of Medicine, Northwestern University, Chicago, USA; 4 Molecular and Cellular Biosciences, University of Massachusetts Amherst, Amherst, USA

**Keywords:** Force, Spring constant, Micropipette, Chromatin, Lamins

## Abstract

**Intro:**

Force measurements of the nucleus, the strongest organelle, have propelled the field of mechanobiology to understand the basic mechanical components of the nucleus and how these components properly support nuclear morphology and function. Micromanipulation force measurement provides separation of the relative roles of nuclear mechanical components chromatin and lamin A.

**Methods:**

To provide access to this technique, we have developed a universal micromanipulation apparatus for inverted microscopes. We outline how to engineer and utilize this apparatus through dual micromanipulators, fashion and calibrate micropipettes, and flow systems to isolate a nucleus and provide force vs. extensions measurements. This force measurement approach provides the unique ability to measure the separate contributions of chromatin at short extensions and lamin A strain stiffening at long extensions. We then investigated the apparatus’ controllable and programmable micromanipulators through compression, isolation, and extension in conjunction with fluorescence to develop new assays for nuclear mechanobiology.

**Results:**

Using this methodology, we provide the first rebuilding of the micromanipulation setup outside of its lab of origin and recapitulate many key findings including spring constant of the nucleus and strain stiffening across many cell types. Furthermore, we have developed new micromanipulation-based techniques to compress nuclei inducing nuclear deformation and/or rupture, track nuclear shape post-isolation, and fluorescence imaging during micromanipulation force measurements.

**Conclusion:**

We provide the workflow to build and use a micromanipulation apparatus with any inverted microscope to perform nucleus isolation, force measurements, and various other biophysical techniques.

**Supplementary Information:**

The online version contains supplementary material available at 10.1007/s12195-022-00734-y.

## Introduction

The nucleus is the stiffest organelle which acts to organize and compartmentalize the genome and its major functions. Recent experiments reveal that loss of nuclear rigidity results in abnormal nuclear shape and rupture which cause dysfunction.^17,30^ These findings clearly link nuclear mechanics back to human diseases, many of which present abnormal nuclear morphology as a diagnostic and prognostic hallmark.^33^

A powerful tool for studies of structure and mechanics of subcellular structures is micromanipulation using glass micropipettes and microneedles, which have been employed to study chromosomes, molecular motors, and other subcellular components.^2,5,15,18,21–23,27,35^ Our own work using this approach to study cell nuclei has provided the novel separation of the two major mechanical elements, chromatin and lamins, commonly disrupted in diseases that present abnormal nuclear shape. Micromanipulation provides isolation of nuclei from living cells and force measurements that reveal a short extension regime, which interrogates chromatin-based nuclear mechanics, while the long extension regime reveals strain stiffening controlled by lamin A.^31^

Physical simulations matched to micromanipulation experiments show that geometry of the nucleus, chromatin, and lamins underlies this phenomena of a short-extension and stronger long-extension regimes.^3,14^ This technique has provided a significant step forward in nuclear mechanics and mechanobiology studies including how histone modification state modulates nuclear mechanics, morphology, and function,^28,29^ how HP1α functions as a chromatin crosslinker in the nucleus and in mitotic chromosomes,^34^ and how the genome is highly crosslinked.^4^ These novel chromatin discoveries are also of interest to chromatin biology.^1^ However, until now, this technique has been inaccessible to other scientists. Here we provide the methodology to build a micromanipulation setup onto any inverted microscope, isolate nuclei, perform force measurements, and outline various other novel biophysical techniques.

## Micromanipulator Setup

**Figure 1:**
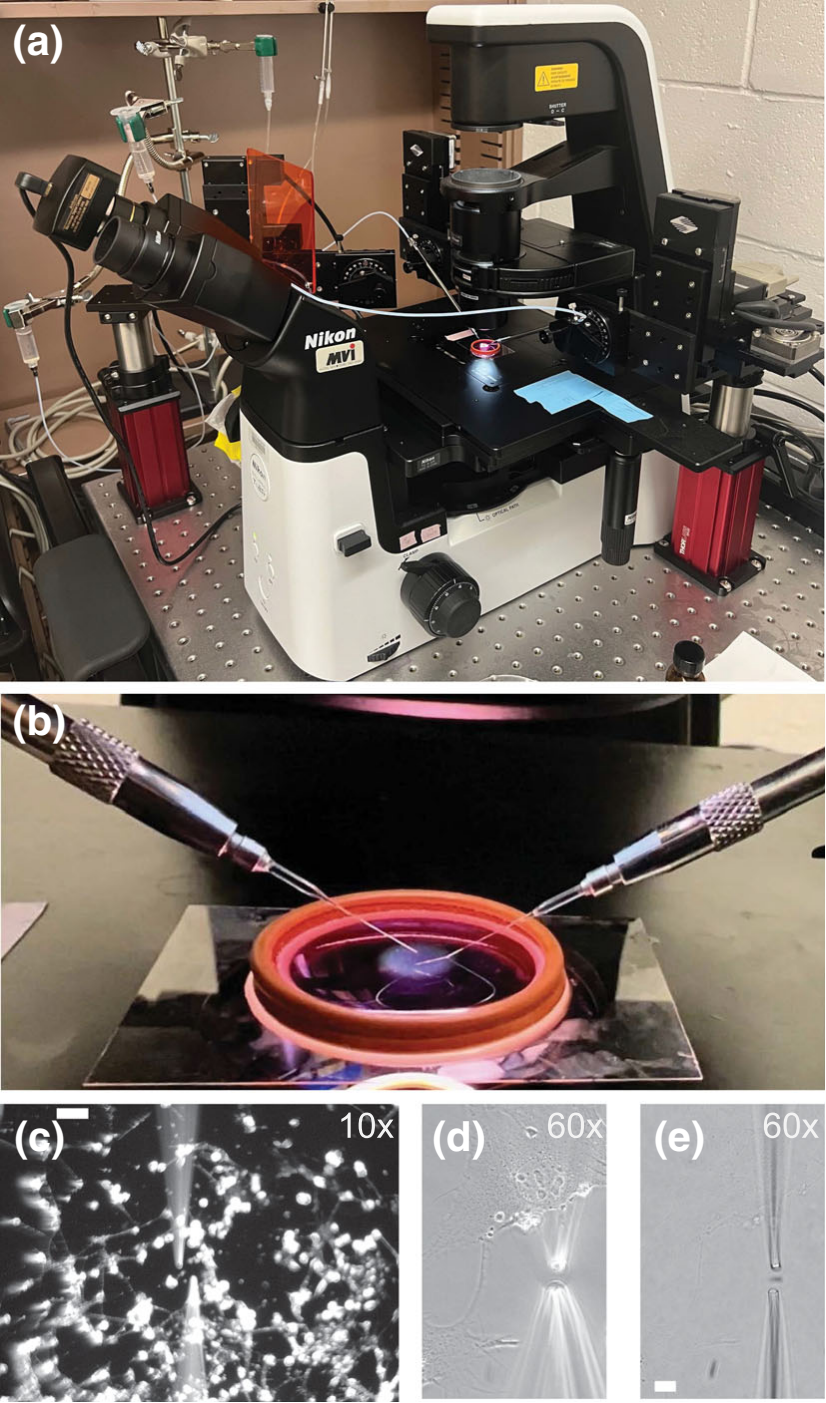
Micromanipulation apparatus setup is adaptable to any inverted microscope. (a) Macro view of the micromanipulation apparatus. Three micromanipulators are placed around the microscope on top of pillars that affix to the air table to position them so that the micropipettes land in the field of view of the microscope. Note the third micromanipulator is not required but can provide flexibility to approaches. Beside the microscope, gravity wells (shown upper left) attached to micropipette holders attached to the micromanipulators to provide flow for the micropipettes. (b) Micro view of the micromanipulation apparatus. Micropipettes entering a coverslip dish of media that contains live cells, which the orange O-ring has a 28 mm diameter for scale. (c–e) Microscope images of micropipettes in a dish with live cells at (c) ×10 magnification where upper left scale bar is 100 µm and (d, e) same image ×60 magnification using (d) Ph3 or (e) Ph1 to focus on the cells or micropipettes respectively, where the bottom left scale bar in e is 10 µm.

The programmable micromanipulators (MP-285, Sutter Instrument, Table 1) with modulable step size were mounted on rigid stands that attach to the air table of any inverted microscope (Fig. 1). A common air table (TMC CleanBench, 30 × 30 or 60 × 36 inches) is required to dampen any vibrations that could be transferred to the micropipette, and thus provides stability. The rigid stands were positioned with a dovetail platform positioned about 1 inch above the microscope stage, 25 mm. The inner corner of the dovetail platform should be roughly 6 inches (150 mm) horizontal and 4 inches (100 mm) backwards from the objective’s field of view. Micromanipulators were mounted to the rigid stand so that they face inwards, and the motor is outwards. To make up this distance to the objective, the micropipette holder was attached to the end of a z vertical and z horizontal extender and set at an angle of 30° (Supplemental protocols). Micropipette holders (Narishige) were loaded with a cut micropipette and placed in the micromanipulator holder for fine-tuned adjustments.

**Table 1:** List of materials.

Item	Company	Item # and link	Function
×2 Motorized micromanipulators programable, use with Z vertical (285,305) and horizontal (285,310) extenders and rod holder (FG-BR-AW)	Sutter Instrument	MP-285	Micromanipulation apparatus
×2 Rigid stands with Platform	Thorlabs	MP100/150/200/250	Micromanipulation apparatus
Micropipette holder and tubing	Narishige	IM-H1	Micromanipulation apparatus
Camera	AmScope	MU130	Micromanipulation apparatus
Microscope vibration control table with screw mounting holes table top	TMC / Ametek	CleanBench	Micromanipulation apparatus
Microscope, with 10X and 60X phase objectives, Ph1 and Ph3 pahse condenser annulus	Nikon	Ts2R-FL	Micromanipulation apparatus
Flaming/Brown micropipette puller P-97 (Sutter Instrument)	Sutter Instrument	P-97	Pulling
Pull/Spray pipettes 6 in, OD 1.0 mm, No Filament	World Precision Instruments	TW100-6	Pulling
Force pipettes 6 in, OD 1.0 mm, Filament	World Precision Instruments	TW100F-6	Pulling
Analog microforge	World Precision Instruments	MF-200	Cutting
Microforge cutting filaments	World Precision Instruments	H3, medium	Cutting
Cutting camera	AmScope	MD130	Cutting (optional)
Base	Thorlabs	BA4	Cutting (pipette holder)
Post holder	Thorlabs	PH	Cutting (pipette holder)
Post insert	Thorlabs	TR	Cutting (pipette holder)
XYZ manual micromanipulator	Thorlabs	DT12XYZ	Cutting (pipette holder)
Mounting adapter	Thorlabs	DT12CTA	Cutting (pipette holder)
Setscrew holding DT12CTA to DT12B from the DT12XYZ set	Thorlabs	SS8S025	Cutting (pipette holder)
Alligator clips	Amazon	Clips	Cutting (pipette holder)
Kite manual micromanipulator	World Precision Instruments	KITE-L/R	Filling
Vacuum pump	Welch	2511 Gemini	Filling
PicoNozzle Kit v1	World Precision Instruments	5430-10	Filling
MicroFil syringe ingection tip 28 gauge, 97 mm long	World Precision Instruments	MF28G-5	Filling
PBS	VWR	PBS	Filling
Triton X-100	VWR	97063-866	Filling
10 mL Syringe with Luer lock	VWR	89215-230	Filling/gravity well
Chemical stand	Cole parmer/VWR	SC-04712-92	Filling/gravity well
Flexible arm clamp	Cole parmer/VWR	EW-08029-06	Gravity well
Low profile cell culture dish	World Precision Instruments	FD3510	Cell culture
FT-S Microforce sensing probe	FemtoTools	FT-S100	Force calibration

The materials used to construct our micromanipulation apparatus. There are many similar versions of each item that can be purchased from many different companies that would provide the same functionality that are not listed here

Optional: An optional helper/third micromanipulator, not necessary to be programmable, can be added to provide micromanipulation support to hold items or to provide a biochemical spray. To decrease bulk and fit around other components, we suggest it be added at a 45 angle on a rigid stand and the use of multiple 2× horizontal extenders to make up distance to the objective field of view.

## Pipette Pulling

A pipette puller (P-97, Sutter Instrument, Table 1) is used to taper capillaries into micropipettes for use in micromanipulation experiments. A glass capillary is loaded into the puller evenly and clamped. Different glass capillaries and their respective program parameters are used depending on the type of micropipette being fashioned. Spray or Pull micropipettes are stiffer and used a 6 in, OD 1.0 mm, No Filament (TW100-6) micropipette with a pull program of Heat 564, Pull 110, Velocity 110, Time 100, and Pressure 500 (for cooling, 1 unit = 1/2 ms). Force micropipettes are more flexible and were fabricated from 6 in, OD 1.0 mm, Filament (TW100F-6) micropipettes with a pull program Heat 561, Pull 220, Velocity 200, Time 20, Pressure 500. Each pull takes roughly 10 s and completes with separation of the capillary into two separate micropipettes (Supplemental protocols and Supplemental movie 1).

## Pipette Cutting

Pipette pullers provide coarse grain size and shape, and further fine cutting is required to shape micropipettes into having a defined opening size. We modified the approach for cutting micropipettes using the MF-200 WPI microforge by constructing a custom micropipette holder/positioner using Thorlabs parts and an alligator clip (Table 1 and Supplemental protocols; original design by M.G. Poirier). This custom micropipette holder allows for coarse positioning in x and y using a base plate that slides easily on bench surfaces. The Thorlabs micropositioner allows for fine adjustment in *x*, *y*, *z* directions to position the micropipette tip in the field of view of the microscope where the cutting filament will be brought to cut. When assembling the cutting apparatus, we loaded the cutting filament in a stage holder to couple the cutting to stage *z* movement but decoupled from the microscope objective focal plane. This allows moving the cutting filament out of focus so that the micropipette can be positioned without collision. When ready to cut, the cutting filament is turned on to a desired heat (determined empirically) and passed through the micropipette using the stage movement to cut the micropipette (Supplemental movie 2). Micropipettes were cut at defined outer diameter sizes of 4–5 µm for pull and force, 6 µm for spray, and 4–10 µm for compression micropipettes (see Fig. 4).

## Pipette Filling

Micropipettes small size taper and opening requires front filling and back filling of biochemicals. Front filling was accomplished by loading the micropipette in a holder attached manual micromanipulator (KITE-L/R, World Precision Instruments) and to tubing connected to a vacuum source (WOB-L® 2511, Welch) to pull liquid through the tip via suction (Table 1). A minute of suction is sufficient to front load the micropipette with liquid. After, a generic 10 mL syringe with Luer tips connected to a long MicroFil tip (WPI, MF28G-5) is used to back fill the micropipette fully. The needle should be placed all the way into the micropipette before back filling to minimize bubbles forming (Supplemental movie 3). This is followed by pointing the micropipette tip down and necessary gentle flicking on the body of the micropipette to remove any visibly trapped air bubbles that would disrupt flow in this system (Supplemental protocols).

## Pipette Loading and Finding

Cut and filled micropipettes were then loaded into a micropipette holder system that provides stability and a controllable flow source (Supplemental movie 4). The IM-H1 Narishige holder system provides attachment to BR-AW micromanipulation clips, a stable holder, and small tubing that can easily be connected to an open syringe to create a gravity well system to control aspiration and spraying through the micropipette (Table 1 and Supplemental protocols). Use of a rubber bulb at the top of the syringe can facilitate flow modulation through this system. However, the micropipette must be first located in the microscopes field of view (FOV) using the micromanipulator controller. Visually the micropipette is aligned with FOV, then lowered into the low profiled cell culture well, located on 10× for centering, and then finally located in the FOV of the 60X object just above the cells and cover glass (Supplemental movie 5).

## Force Calibration

A primary calibration micropipette with a known spring constant is required for the methodology used in creating all other calibrated micropipettes for force measurement. We used a microforce sensor (FT-S100, FemtoTools, Table 1) that converts force to voltage in a standardized manner to calibrate a primary calibration micropipette. We programmed movement of the primary calibrated force micropipette to move 10 and 50 µm while in contact with the microforce sensor. This process was repeated in triplicate recording the voltage at maximum compression. The purpose of the sensor is to translate the force of the calibration micropipette into a voltage; the voltage can then be converted back into a force given the volt per force of the sensor. By pressing the calibration micropipette into the sensor at specific and repeatable amounts, the spring constant of the calibration micropipette can be derived as force (given by the sensor) divided by distance (given by the preprogrammed distance for the micropipette to move). A custom program was written in Labview for calculations (available upon request). For nuclear mechanics we suggest a primary calibration micropipette of 1.5–2 nN/µm.

Force micropipettes are cut to a defined size of 4–5 µm and spring constant of 1.2–2 nN/µm. We load a primary calibration micropipette with known spring constant force micropipette (determined above) and a newly cut force micropipette with an unknown spring constant. The unknown force micropipette is held stationary while the known is brought into contact with a set overlap length of 15 µm. This is the initial position. Next the known micropipette is programmed to move 6 µm along the x-axis to push against the unknown micropipette via the micromanipulator at 400 nm/s and then hold. This position is the resistance position. Finally, the unknown force micropipette is moved in the y-axis away quickly to withdraw it. The known micropipette then jumps to its unresisted position now that it is not held back by the other unknown micorpipette. If both micropipettes are of equal strength, then after the known micropipette is told to move 6 µm, both micropipettes will be deflecting the same amount 3 µm. If the resistance position is less than the unknown micropipette is stronger, if greater then unknown is weaker (resistance—initial) divided by (final—resistance) multiplied by known spring constant.

## Nuclear Isolation and Force Measurements

**Figure 2:**
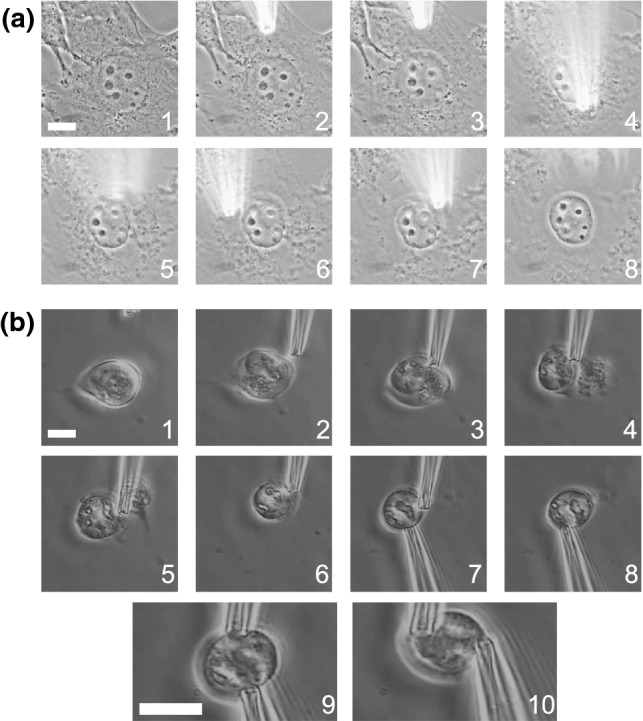
Micromanipulation excels at isolating a single nucleus from a live cell. The cell nucleus can be isolated by breaking open the cell with mild detergent 0.05% triton X-100. (a) Mouse Embryonic Fibroblasts null for vimentin (MEF V−/−) nuclei are easily isolated without the need of actin depolymerization. (b) Wild type Mouse Embryonic Fibroblasts nuclei can be isolated from cells treated with latrunculin A actin depolymerizing agent after 45 min of treatment. Images 1–6 show a spray micropipette isolating the nucleus. Images 7–8 shows the grabbing of the nucleus by the pull micropipette (bottom) and removal of the spray micropipette (top). Bottom row of images (9–10) shows an isolated nucleus being prepared for micromanipulation force measurement by force micropipette (top) and pull micropipette (bottom). The scale bar is 10 µm.

Micromanipulation can isolate nuclei from living cells in a few minutes. Our most used cell line is vimentin null (MEF V−/−) which provides easy isolation without the need for actin depolymerization and provides similar cell biological and mechanical measurements^28,31^ (Fig. 2a). Most other cell types can be treated with 1 µg/mL latrunculin A for 45 min before isolation to depolymerize actin to allow for nucleus isolation (Fig. 2b). A micropipette sized to 4–5 µm tip is loaded with mild detergent (triton X-100 0.05%) for nucleus isolation. This micropipette is located in 10× objective before moving to 60× oil objective for finer control of the micropipette relative to the nucleus. The gravity well is lifted to expel the mild detergent and break open the plasma membrane (Supplemental protocols). A second micropipette of similar size is loaded with PBS for capture of the isolated nucleus. This micropipette aids isolation by pulling the nucleus out of cell remnants and serving as the future pull micropipette. The spray micropipette is removed and replaced with a precalibrated force micropipette in preparation for force measurements.

Use of dual micromanipulation allows for controlled extension of a single isolated nucleus. Isolation and attachment to one micropipette (aka pull micropipette) is followed by adding a second force calibrated micropipette (aka force micropipette) attaching to the opposite side of the nucleus. Attachment is accomplished by dropping the gravity well to provide aspiration of the nucleus into the micropipette which causes non-specific attachment of the nucleus to the glass of the micropipette after which the gravity well is moved back to neutral. The force micropipette is tracked while the pull micropipette is moved into position so that each micropipette is parallel, and the nucleus is set for extension. After moving into position, the pull micropipette is moved to relieve any pulling or pushing.

**Figure 3:**
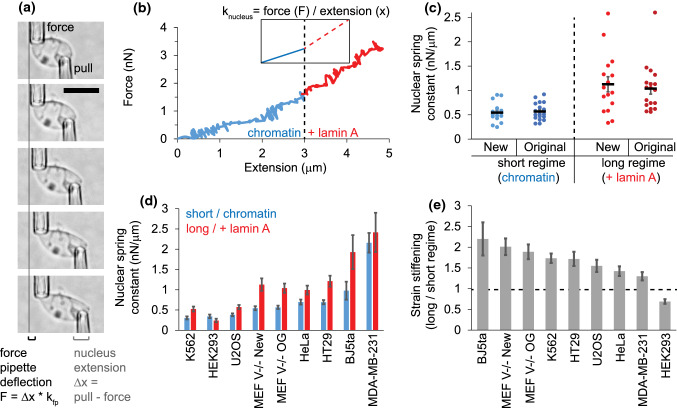
The micromanipulation apparatus recapitulates the main findings of force measurements across cell types, chromatin base and lamin A strain stiffening. (a) Images from a force extension experiment showing the pull micropipette (bottom right) extending the nucleus (change in distance between micropipettes) and the deflection of the force micropipette (top left) which multiplied by its precalibrated bending constant (*k*_fp_) provides a measure of force (*F*). The scale bar is 10 µm. (b) Force extension plot of data from panel a showing the separate regimes of short, dominated by chromatin (blue), and long, chromatin plus strain stiffening from lamin A (red). (c) Comparison showing the short and long regime values for the new apparatus (*n* = 16) to the original setup (*n* = 18), where short is the chromatin-dominated regime (blue) and long is chromatin + lamin A regime (red). (d) Micromanipulation force measurements are adaptable to nuclei of all types of cells. Graphed in order of weakest to strongest short extension regime (blue), with long regimes (red) also shown. (e) All cell nuclear force measures graphed for strain stiffening (long/short regime) from greatest to least. (K562 *n* = 4; HEK293, *n* = 16; U2OS, *n* = 11; HeLa, *n* = 13; HT29, *n* = 19; BJ5ta, *n* = 4; MDA-MB-231, *n* = 6). Data for MEF V−/− new, K562, and MDA-MB-231 are novel measurements to this paper. Data for HEK293, HeLa, MEF V−/− original, HT29, and BJ are reanalyzed from Ref. 31, while U2OS data came from Ref. 34. Supplemental Table 1 provides the raw numbers for generating panels d and e.

We tested whether this new system performs similarly to the original system. Single nuclei were subjected to controlled micromanipulation extension at 50 nm/s by moving the pull pipette and tracking the position of both the force and pull micropipettes. The deflection of the force micropipette multiplied by precalibrated spring constant provides a measure of force (*F* = Δ*x* * *k*_fp_) while the change in distance between micropipettes tracks nucleus extension (Δ*x* = pull − force pipette position; Fig. 3a). The slope of force/extension (nN/µm) line provides the spring constant of the nucleus (example force measurement calculation in Supplemental protocols). The new setup force vs. extension graphs recapitulate the two-regime force response with an initial spring constant measured by slope at < 3 µm (blue, ~ 30% strain) which switches to a stiffer regime at extensions > 3 µm (red; Fig. 3a and 3b). The new apparatus provided similar average force measurements of MEF V−/− for short (chromatin) and long (chromatin + lamin A) regimes compared to the original apparatus^31^ (Fig. 3c). The micromanipulation apparatus has provided the ability to measure nuclei in many different cell types providing comparison of relative strengths (Fig. 3d, Supplemental Table 1). Overall, all nuclei with high ratios of lamin A to lamin B produce a strain stiffening at long extensions, except HEK293 which have low levels of lamin A (Fig. 3e;^31^), in agreement with previous reports.^36^

## Nuclear Compression *via* Micromanipulation

**Figure 4:**
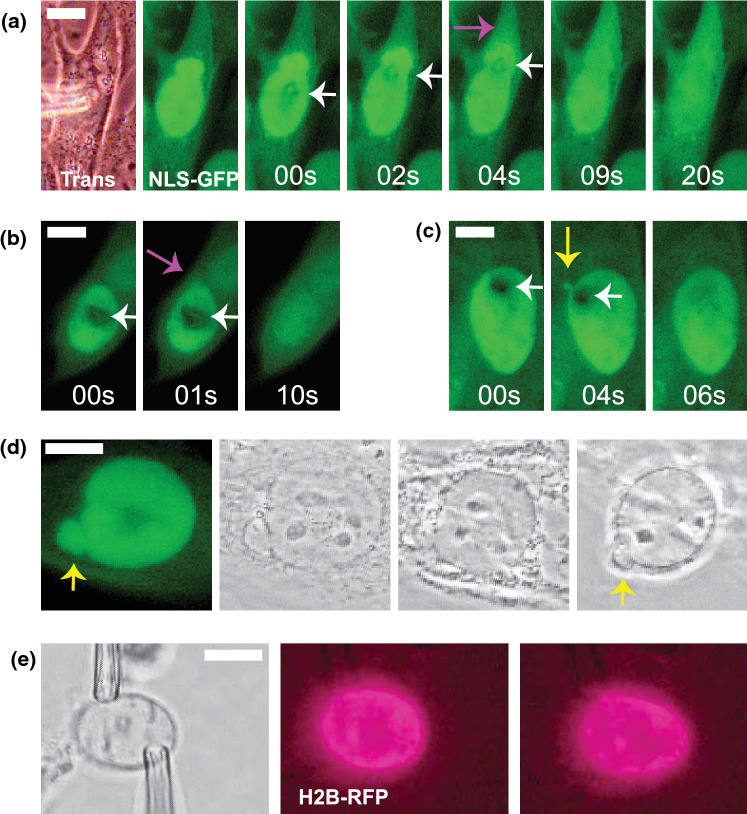
Micromanipulation apparatus is adaptable to many different biophysical assays. Micromanipulation can be used to cause moving compression (white arrow) of the nucleus while imaging NLS-GFP to cause (a) bleb-based or (b) non-bleb-based nuclear rupture (purple arrow) and (c) temporary bleb formation (yellow arrow). Using a precalibrated force micropipette has the capability of measuring applied force during compression. (d) Micromanipulation-based nucleus isolation can be used to assay the persistence of nuclear morphology and blebs (yellow arrow) post removal from the cell. (e) Widefield fluorescent imaging of histones *via* H2B-RFP during micromanipulation force extension measurement. Proof of principle that fluorescence imaging can be coupled to force-extension measurements to track organization, stretching, and movement of key nuclear components. The scale bar is 10 µm.

Fine control of micropipettes provides the ability to assay nuclear resistance to moving compression. Force calibrated stiffer micropipettes (5 nN/µm) sized 4–10 µm tip were used to either compress the nucleus or compress with horizontal motion to the edge of the nucleus while imaging nuclear compartmentalization *via* nuclear localization signal green fluorescence protein (NLS-GFP, Figs. 4a–4c). Our novel data reveals that simple local compression of nuclei does not compromise the nuclear envelope, while local moving compression resulted in substantial increase in nuclear ruptures from blebbed or normally shaped nuclei (Figs. 4a, 4b). Furthermore, generating compression with micropipettes can temporarily induce small blebs, though they reabsorb rapidly (Fig. 4c). This ability to modulate local compression with or without motion could be leveraged to investigate nuclear ruptures as well as nuclear bleb formation.

## Micromanipulation-Based Single Nucleus Isolation is Adaptable to Many Approaches

Nucleus isolation can provide a fresh, gently isolated nucleus for many single nucleus/cell studies. The nucleus can be held with one micropipette while the second or optional third micropipette could be loaded with any biochemical to spray onto the nucleus for biochemical assays. Furthermore, isolation of a single nucleus can be used to probe nucleus elastic or plastic properties outside of the cell through measuring nucleus size and shape. For example, here we show novel data that nuclear blebs are persistent/plastic deformations that remain upon nucleus isolation from the cell (Fig. 4d), which agrees with previous work showing cutting of the cell did not relieve abnormal nuclear shape.^38^ This technique can be adapted to any number of new approaches requiring a freshly and gently isolated nucleus.

## Visualizing Nuclear Organization During Micromanipulation Force Measurements

The ability to build the micromanipulation apparatus onto any microscope allows for greater exploration of imaging alongside force extension measurements. Previously, micromanipulation experiments on mitotic chromosomes have benefited greatly from coupling to imaging approaches to see the finer detailed organization of chromosomes.^5,35^ This apparatus was built onto a basic widefield imaging system. We provide proof-of-principal imaging for H2B-RFP (Invitrogen, CellLight) during micromanipulation force measurements (Fig. 4e). There are many possible approaches for imaging of bulk chromatin, chromosome territories, and specific loci in conjunction with micromanipulation.

## Conclusion

Micromanipulation force measurements have provided a novel leap forward in nuclear mechanics studies. Each force measurement technique has found its niche in mechanobiology. Optical tweezers in yeast, that do not have lamins, provide probing of chromatin-peripheral tethering and chromatin^26,41^ while in mammalian cells probe small local deformations.^11^ Micropipette aspiration provides a strong measure of lamin-based nuclear mechanics through high local strain > 100%^6,12,20,36,40^ which has provided much of what we know about lamins. Atomic force microscopy provides general changes in nuclear strength through perturbations of either chromatin^9,10,19^ or lamins.^25,39^ However, none of these techniques could separate chromatin and lamin mechanical contributions. Micromanipulation provides controlled deformation where both force and extension could be measured simultaneously that was necessary to separate chromatin and lamin regimes.^3,31^ Recently, the ability to finely track nuclear deformation and force *via* combined atomic force microscopy and light sheet imaging has recapitulated the separation of chromatin-dominated short deformation regime and lamin A/C-based stiffening at higher deformation.^13^ These direct measures of the separate chromatin and lamin regimes agree with data provided indirectly by many other force measurement techniques and cell biology approaches.^32^ We have provided the ability to build and use a micromanipulation force measurement apparatus on any inverted microscope so that more researchers can access the ability to directly measure the separate contributions of chromatin and lamin A in their systems and experiments.

Micromanipulation apparatus provides novel approaches to expand the field of mechanobiology beyond nuclear force-extension measurements. Use of nuclear confinement approaches has led to recent discoveries in nuclear deformations, blebbing, and ruptures that cause nuclear dysfunction.^8,16,24^ Micropipette aspiration has been used to induce nuclear rupture loss to show rupture causes dysfunction^16^ and to connect amount of pressure applied to loss amount and rates.^42^ Here we outline nuclear compression with micropipettes providing the ability to induce long and short-lived blebs and induce nuclear ruptures to aid furth investigation of nuclear rupture dynamics, forces, and consequences to nuclear function. These approaches will be needed to keep pace with screens revealing more determinants of nuclear shape.^37^ Finally, combining nuclear fluorescence imaging during micromanipulation could provide a means to calibrate future force probes, as some lamin and lamin-chromatin force probes are already developed.^7^ Micromanipulation-calibrated fluorescence FRET force probes would not only allow researchers to measure force changes *via* fluoresce change in live cells in cell culture or *in vivo* systems but also separate chromatin and lamin A force regimes.

## Supplementary Information

Below is the link to the electronic supplementary material.

Supplementary file1 (PDF 162 kb)

Supplementary file2 (MOV 20342 kb)

Supplementary file3 (MP4 203901 kb)

Supplementary file4 (MP4 189522 kb)

Supplementary file5 (MP4 79263 kb)

Supplementary file6 (MP4 27172 kb)

Supplementary file7 (DOCX 12103 kb)

Supplementary file8 (XLSX 27 kb)

Supplementary file9 (DOCX 16 kb)

## Abbreviations

MEF: Mouse embryonic fibroblast
V -/-: Vimentin null
NLS-GFP: Nuclear localization signal green fluorescence protein

## Citation diversity statement

Recent work in several fields of science has identified a bias in citation practices such that papers from women and other minority scholars are under-cited relative to the number of papers in the field. We recognize this bias and have worked diligently to ensure that we are referencing appropriate papers with fair gender and racial author inclusion.
