# Supplementary material for: A Versatile Micromanipulation Apparatus for Biophysical Assays of the Cell Nucleus
Source: Cell Mol Bioeng. 2022 Sep 6;15(4):303–12. doi: 10.1007/s12195-022-00734-y (PMC9474788; doi:10.1007/s12195-022-00734-y)
Supplement: Supplementary file 1 — Supplementary file1 (PDF 162 kb) [file 12195_2022_734_MOESM1_ESM.pdf]

Function
Micromanipulation apparatus
Micromanipulation apparatus
Micromanipulation apparatus
Micromanipulation apparatus
Micromanipulation apparatus
Micromanipulation apparatus
Pulling
Pulling
Pulling
Cutting
Cutting
Cutting (optional)
Cutting (pipette holder)
Cutting (pipette holder)
Cutting (pipette holder)
Cutting (pipette holder)
Cutting (pipette holder)
Cutting (pipette holder)
Cutting (pipette holder)
Filling
Filling
Filling
Filling
Filling
Filling
Filling / Gravity well
Filling / Gravity well
Gravity well

Cell Culture
Force calibration
