# Supplementary material for: A Versatile Micromanipulation Apparatus for Biophysical Assays of the Cell Nucleus
Source: Cell Mol Bioeng. 2022 Sep 6;15(4):303–12. doi: 10.1007/s12195-022-00734-y (PMC9474788; doi:10.1007/s12195-022-00734-y)
Supplement: Supplementary file 7 — Supplementary file7 (DOCX 12103 kb) [file 12195_2022_734_MOESM7_ESM.docx]

Supplemental protocols

This is a supplemental document for the manuscript “A versatile micromanipulation apparatus for biophysical assays of the cell nucleus” at the journal Cellular and Molecular Bioengineering.

# Pulling

- 1. Sutter Instruments Flaming/Brown micropipette puller P-97 is used to taper capillaries to produce micropipettes for use in micromanipulation experiments.
  2. A glass capillary is loaded into one side of the puller and clamped. Hold the two clamps together with one hand, as the other side clamp is fastened with the other hand. Note: it is best to make a mark on the starting clamp with a marker where the end of the glass capillary should be so that when fully loaded the capillary is centered.
  3. Different glass capillaries and their respective program parameters are used depending on the type of micropipette being fashioned.
     - Spray and Pull pipettes, 6 in, OD 1.0 mm, No Filament (TW100-6) program parameters
       - Heat 564
       - Pull 110
       - Velocity 110
       - Time 100 (for cooling, 1 unit = ½ milliseconds)
       - Pressure 500
     - Force pipettes, 6 in, OD 1.0 mm, Filament (TW100F-6) program parameters
       - Heat 561
       - Pull 220
       - Velocity 200
       - Time 20 (for cooling, 1 unit = ½ milliseconds)
       - Pressure 500
  4. Each pull takes roughly 10 seconds and completes with separation of the capillary into two separate micropipettes.

- Supplemental movie 1 provides an example of micropipette pulling -

# Cutting

Pipette pullers provide coarse grain size and shape pipettes that require cutting to make an open pipette tip with a defined size. We modified the approach for cutting micropipettes using the MF-200 WPI microforge and a custom pipette holder/positioner using Thorlabs parts.

- 1. Camera MD130 AmScope (optional)
  2. Customized cutting pipette holder (see picture on right)
     - BA4 base
     -
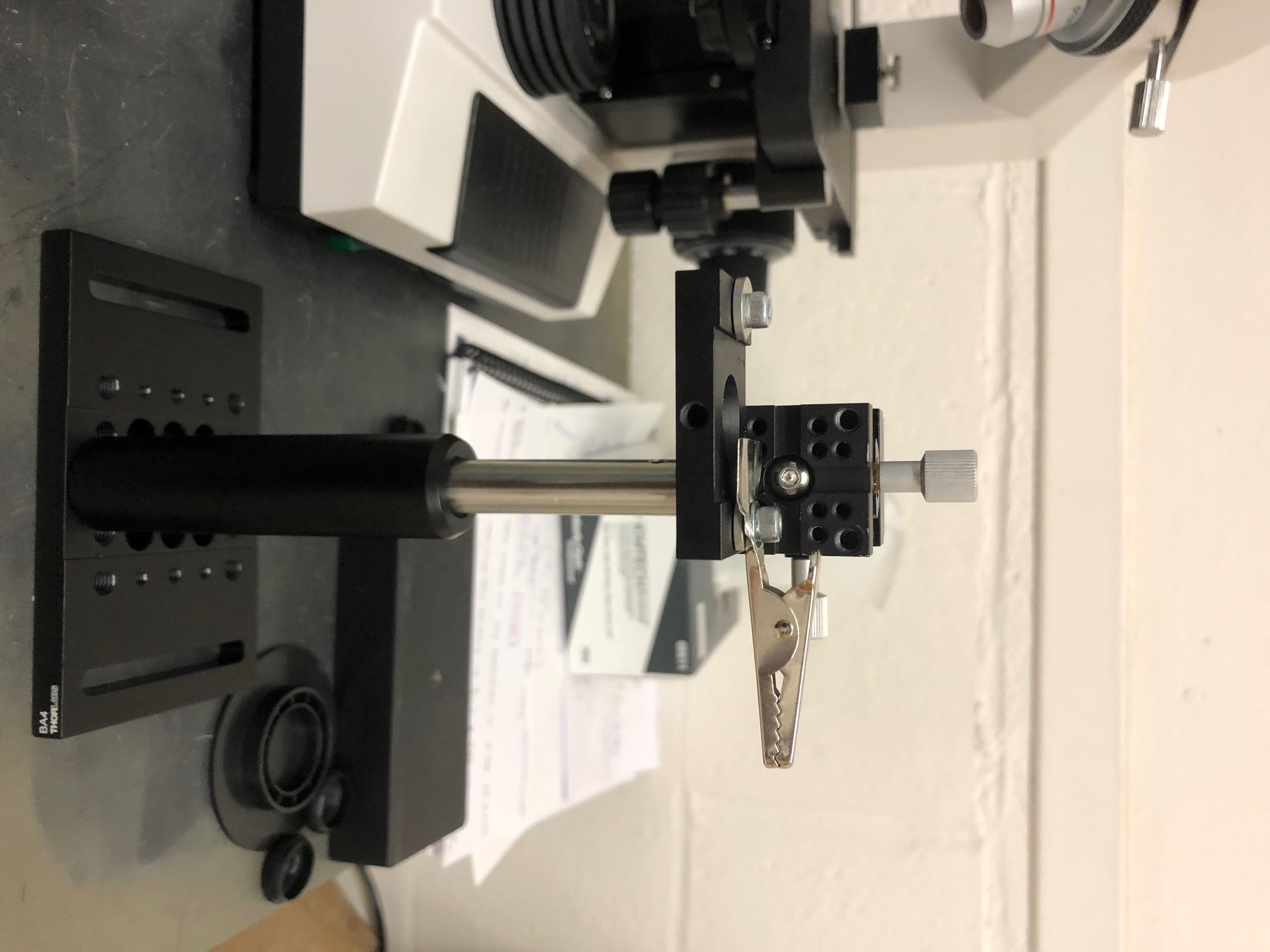
PH post holder
     - TR internal post
     - DT12 XYZ micromanipulators
       - Remove DT12B from bottom and attach to DT12CTA via 8/32 setscrew
       - It is best to take apart the Z manipulator and the right angle adapter and point it upwards of the XY manipulators
     - Top plate adapter DT12CTA
     - Alligator clip
     - Filaments (medium H3 WPI)
     - MF-200 WPI microforge
     - Construction flow of cutting pipette holder: Base → post (has screw on top) → post insert → XYZ manipulator on face with all the different shaped holes in it → mounting adapter DT12CTA side of Z manipulator → setscrew DT12B → alligator clip
       -
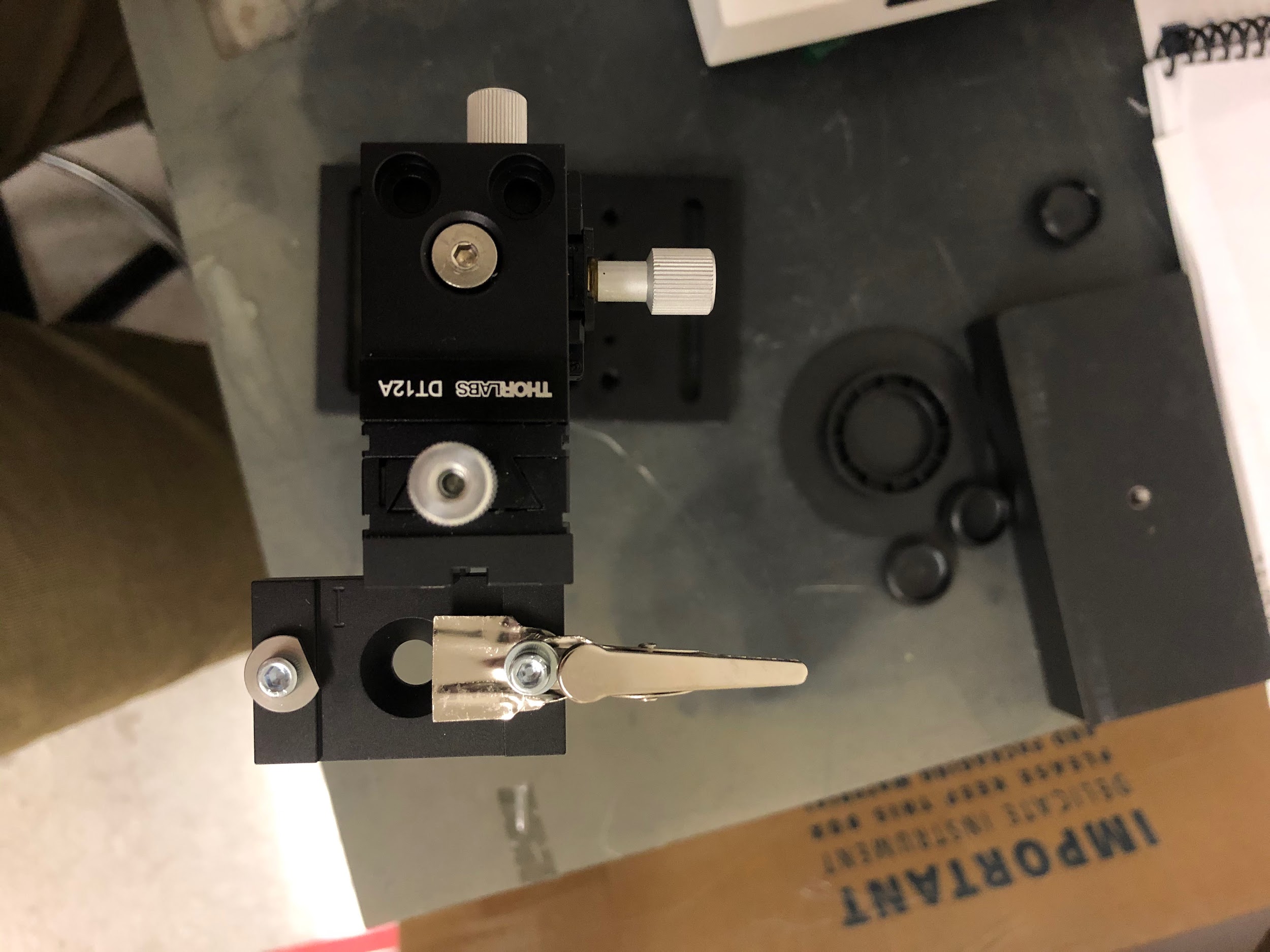
This setup is beneficial because the pipette holder system has XYZ adjustment capabilities allowing for fine movements so the pipette tip can be brought into focus and the correct cutting placement without having to adjust the filament placement or other more stationary aspects of the cutting setup.
       - It also allows for coarse movements once the pipette is in the cutting area instead of sliding the base of the cutting holder along the table or resetting the placement of the pipette.
     - Alligator clip design
       - Wedge pliers into the back tube of the alligator clip. Open the pliers to start opening the tube. Once the tube is opened, use the pliers to push apart the two sides. Alternate between spreading the tube edges and using a hammer to push it down.
       - Once the tube is completely flattened, use a screwdriver to push into the pre-punched hole to make it larger. Start with a smaller diameter screwdriver and then work up to a bigger one. Then use pliers, tweezers, or scissors to shape the hole.
       - Check the size of the hole by pushing through the screw that will be used to attach the clip to the rest of the setup (connects to the DT12B)
       - Once the hole is the correct size, use the washer that fits into the hole of the DT12B, then put down the alligator clip, a flat washer, then a screw going through all of this.
       - I set up the alligator clip so that it runs perpendicular to the short edge of the DT12B. This made it so that the base of the set up does not interfere with the microscope base and the pipette can be put into position easily.
  3. The filament must have a bubble on it to help in the cutting process. To do this, take a pipette tip (hold in the cutting holder), get in focus, and bring the filament up. With a low - medium level of heat, allow some of the pipette tip to melt onto the filament to create a glass bubble on the filament.
     - Cutting is done with the heat at an empirically determined setting. The pipette is lined up with the bubble of the filament where you want to cut the pipette.
     - The filament is raised and lowered in one smooth motion to make a cut then return to its original position. Once the pipette is cut, remove the pipette, raise the filament into focus, and turn on the heat to 100 (max setting) to melt off any excess glass.
     - Troubleshooting
       - If the filament gets bent, the cleaning rod of the Narishige pipette holder can be used to bend the filament back into shape.
       - The cutting point of the pipette is important because if we are cutting too close to the tip the opening will be too small to properly spray or grab the nucleus. If we try to cut too high, the heating filament (which is really malleable) can get bent because the pipette will bounce off of the filament instead of the filament going through the pipette
       - If the filament is too hot, the tip of the pipette can will seal closed from cutting.
       - if the filament is left in contact with the pipette for too long the pipette can close
       - If the filament is not hot enough there will be a deflection behavior off the filament, and not a clean cut of the pipette.
     - After pipettes are cut, they can be measured in the AmScope and ImageJ programs.

- Supplemental movie 2 provides an example of micropipette cutting -


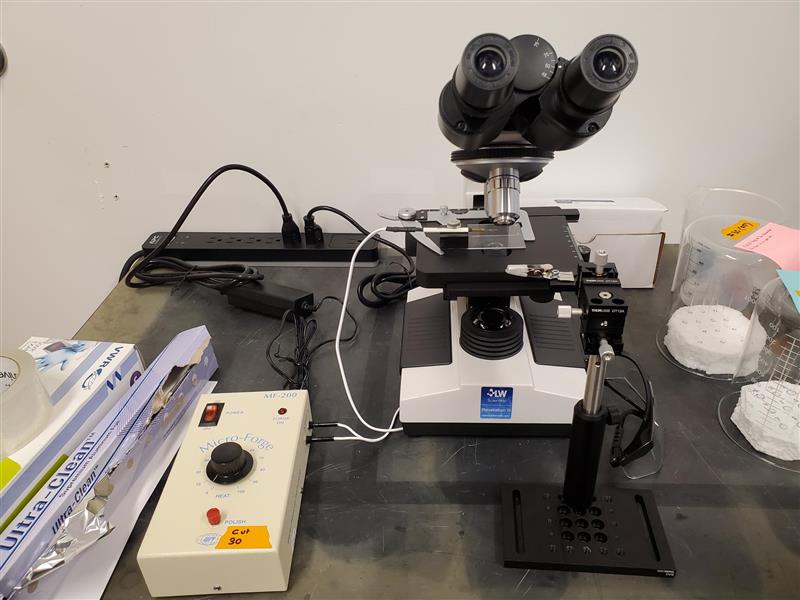


#
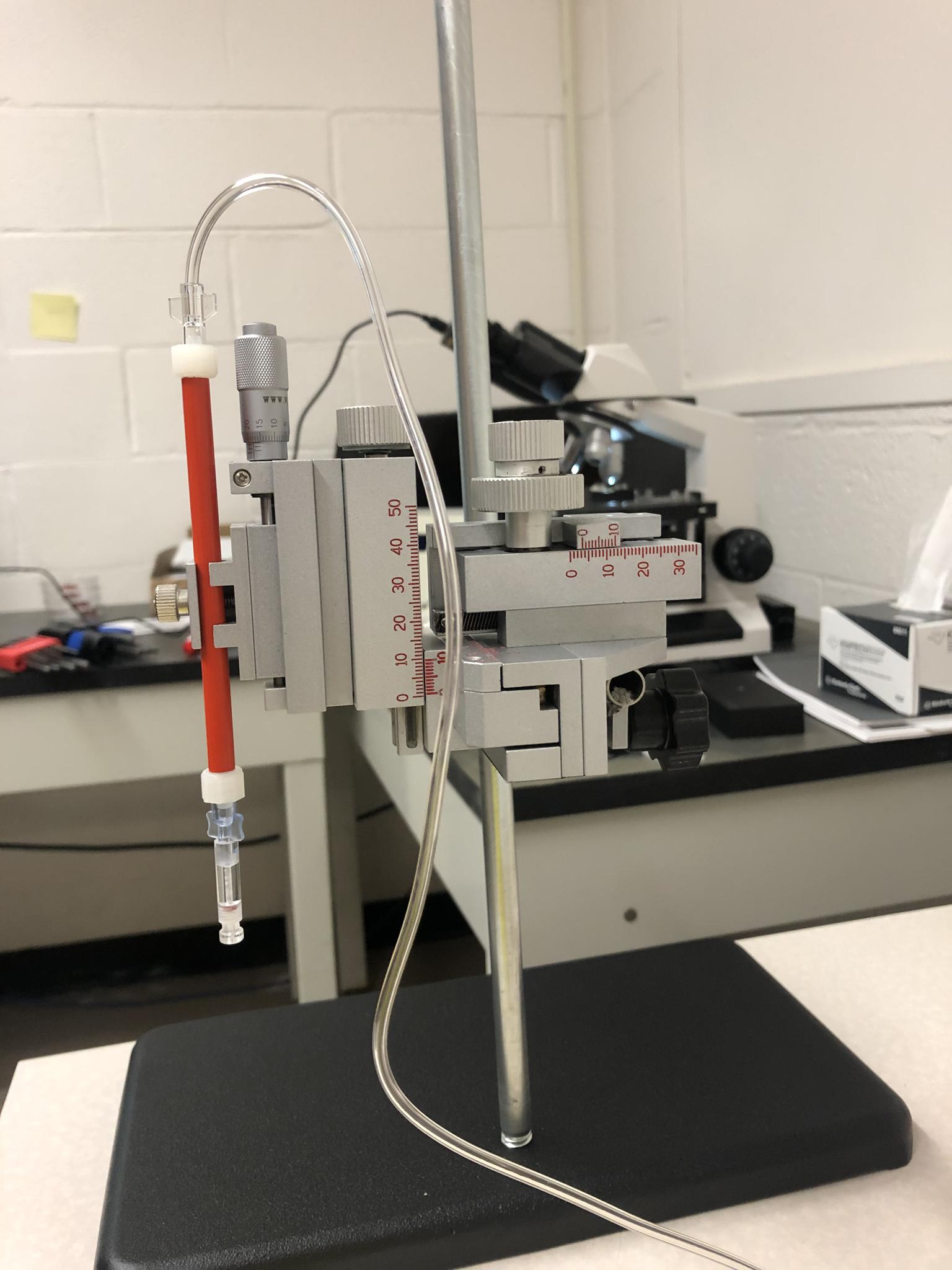
Filling

After cutting the micropipette’s still small opening requires first front filling via suction and then back filling with a syringe and flexible needle.

- 1. Front fill
- Insert the cut pipette into red pipette holder (PicoNozzle Kit v1) and tighten by screwing the piconozzle.
- Position an open 1.5 mL epi tube full of desired biochemical underneath the front filling station, then carefully lower the pipette using the Z manipulator
- Once the tip of the pipette is submerged into the tube, turn the vacuum system on and let it run for about 1-2 minutes.
- Turn the vacuum off, raise the pipette back out of the cap, loosen the pipette holder, and take out the pipette so that you can backfill it.
-
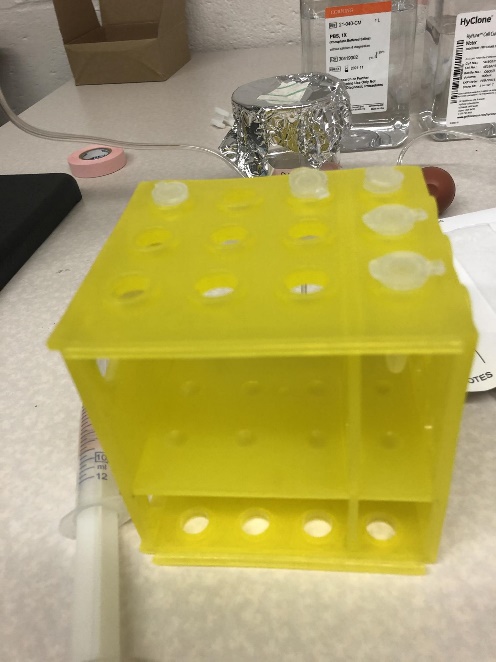
To double check that the front fill was done completely, you can hold the pipette up to the light and make sure that the pipette tip has liquid in it in the entire tapered part.
  1. Backfill
     - After the pipette has been front filled, we need to backfill using a 28 guage 97 mm MicroFil tip + 10 mL syringe with luer lock with the same biochemical that you have front filled the pipette with.
     -
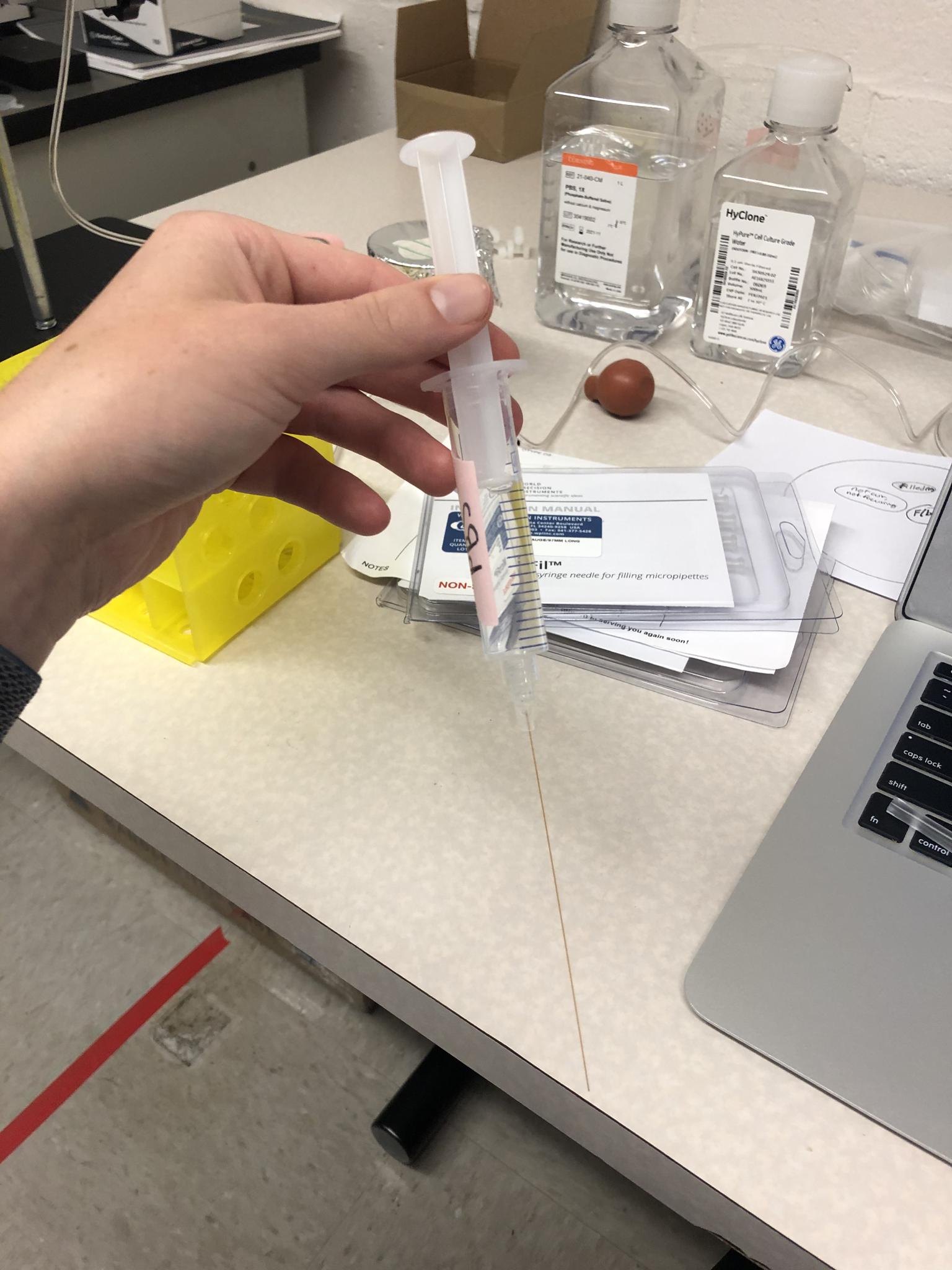
After raising the red pipette holder and moving the yellow test tube holder out of the way, loosen the red pipette holder and pull the pipette out.
     - Hold the pipette up to the light and make sure that the solution has been front filled into the pipette tip.
     - Insert the syringe with the same biochemical into the back end of the pipette and finish filling via backfill.
     - Air bubbles will form in between the front and back filled sections. To tap out the air bubbles, hold the pipette between the pointer and thumb, with the middle finger behind to act as a backstop. Use the pointer finger on the other hand and flick the pipette so that it hits the middle finger backstop with each flick once the air bubbles have travelled all the way up and reached the top of the pipette, the pipette is ready to be loaded and used.

- Supplemental movie 3 provides an example of micropipette filling -

# Micromanipulators

Micromanipulators are attached directly to the air table via rigid stands and not the microscope to decouple from any movements.

- 1. Pillars (MP100, 150, 200, or 250 [Thor Lab Rigid Stands](https://www.thorlabs.com/thorproduct.cfm?partnumber=MP200)) are used station the micromanipulators on the air table separate from the microscope. This allows them to be supported by the air tables vibration canceling and decouples them from any movement in the microscope that may cause mechanical vibration or noise.
  2.
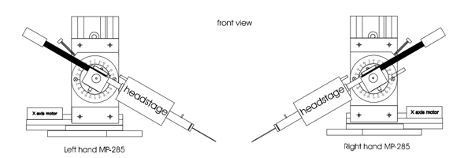
2X Micromanipulator with Control box ([MP-285A](https://www.sutter.com/MICROMANIPULATION/mp285.html) Sutter) provides a fully programmable micromanipulator that is reliable with 40 nm steps and modulation of steps per second (or pull rate). Images from the [Sutter Instrument MP-285 Reference Manual](https://www.sutter.com/manuals/MP-285A_RefMan.pdf).
     - Face plate (comes with the micromanipulators)
     - Extender bracket
     - Rotating dovetail
     - Headstage
     - Pipette holder FG-BR-AW (listed on site as Rod holding clamp for XenoWorkd Injector)
       - Needed to hold the Narishige micropipette holder
     - Extensions to gain room away from MMs and over microscope stage
       - Z axis vertical extension 285305
         1. Gets added first
       - Z axis horizontal extension 285310
         1. Gets added second
  3. Micropipette holder set ([IM-H1](http://products.narishige-group.com/group1/IM-H1/injection/english.html) Narishige) holds the pulled and cut micropipettes and seamlessly attaches to gravity wells (see next section) to control flow rate through the micropipette.
     - HI-7 injection holder
     - CT-1 PTFE tubing
     - CI-1 tube connector.
  4. Description of set up
     - The pillars were set up close to the base of the microscope while allowing for full stage mobility. With this placement, the platform on the pillars was moved so that the end of the platform was roughly in line with the objectives. This made it so that when the faceplate is loaded onto the platform it holds the micropipettes in line with the objectives
     - The angles for the holders are shown in images below. On this item there is an indicator shaped like a whale tale (blue arrows pointing at this indicator in the first two images). I used these to determine the angle. Another way to measure the angle of the pipette is to use a level. I used the iPhone app “Measure”. Then, I laid my phone along the pipette holder to determine what angle the pipette is at.
     - See pictures below of angles on our apparatus

– Supplemental movie 4 and 5 provide example loading and finding of micropipettes –

*** Other images of the apparatus are included in the main manuscript Figure 1.


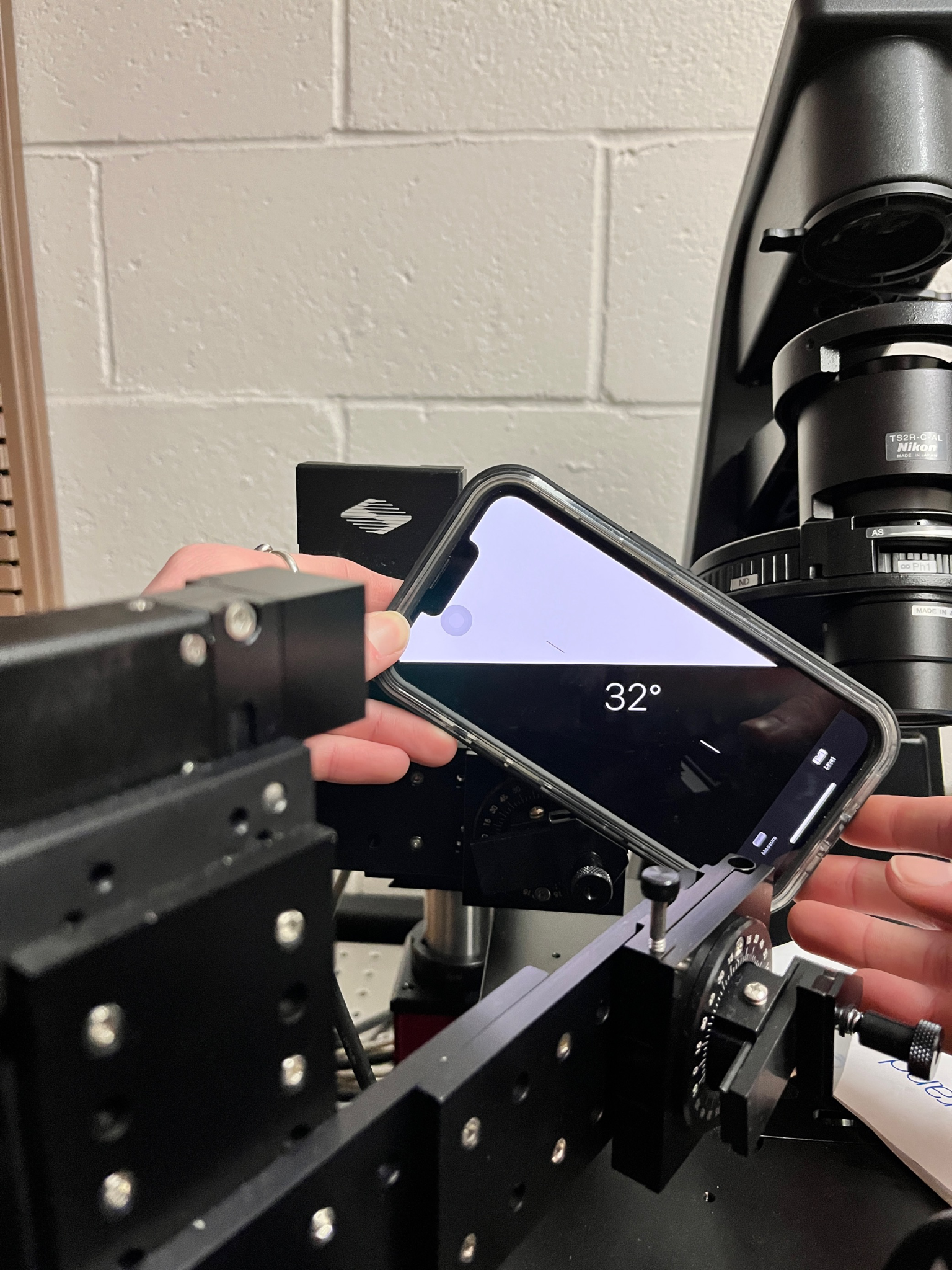

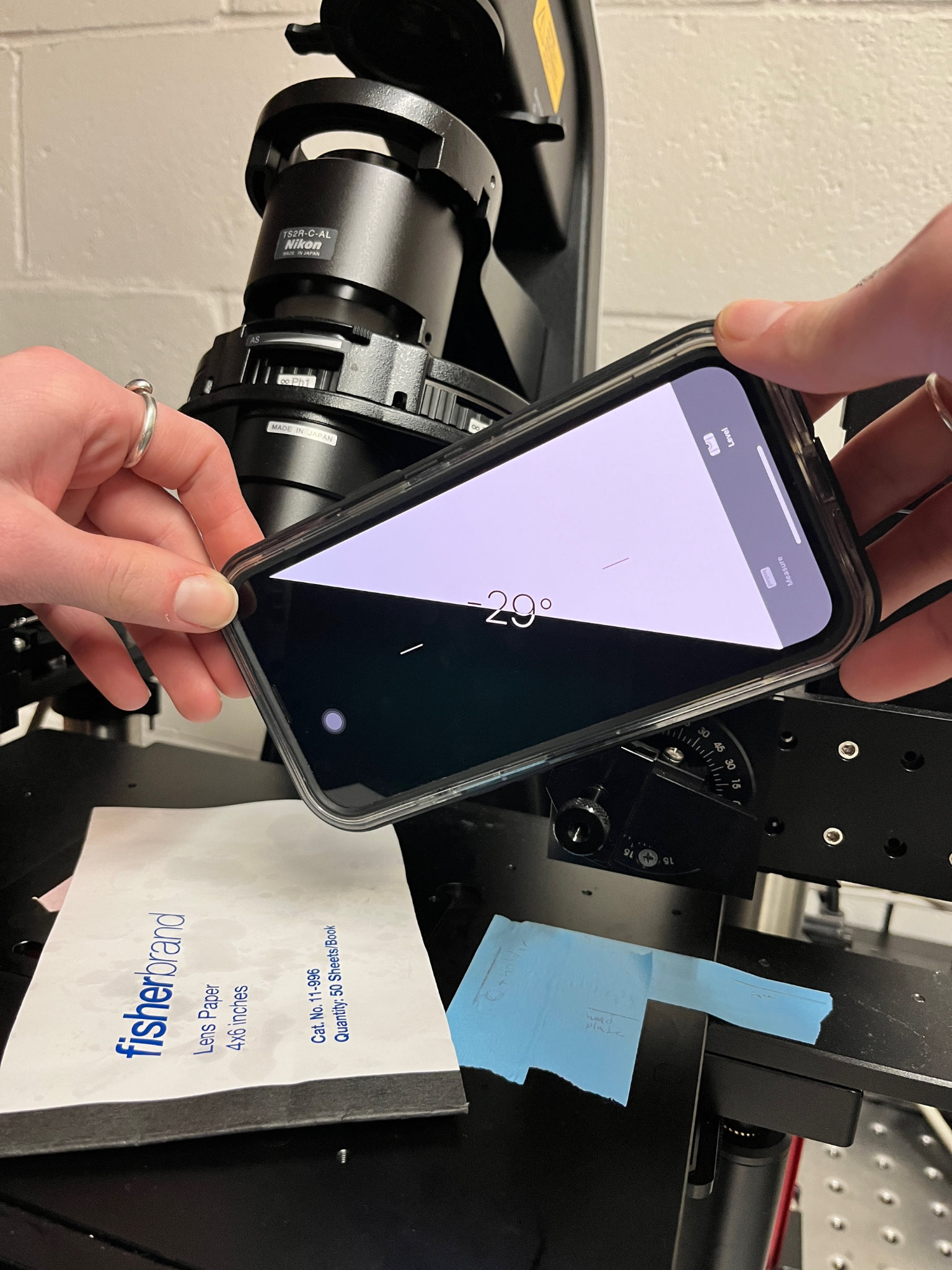


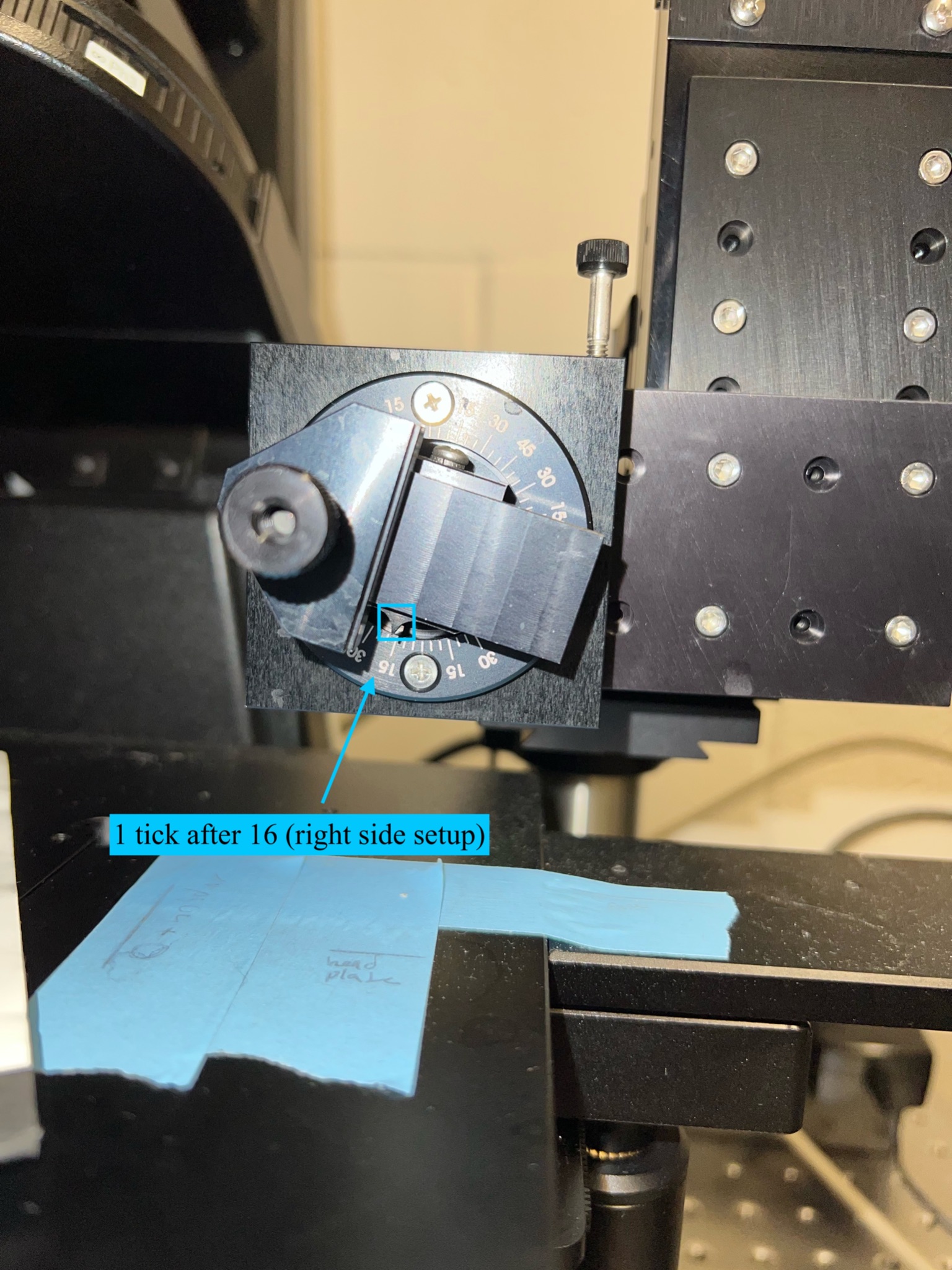

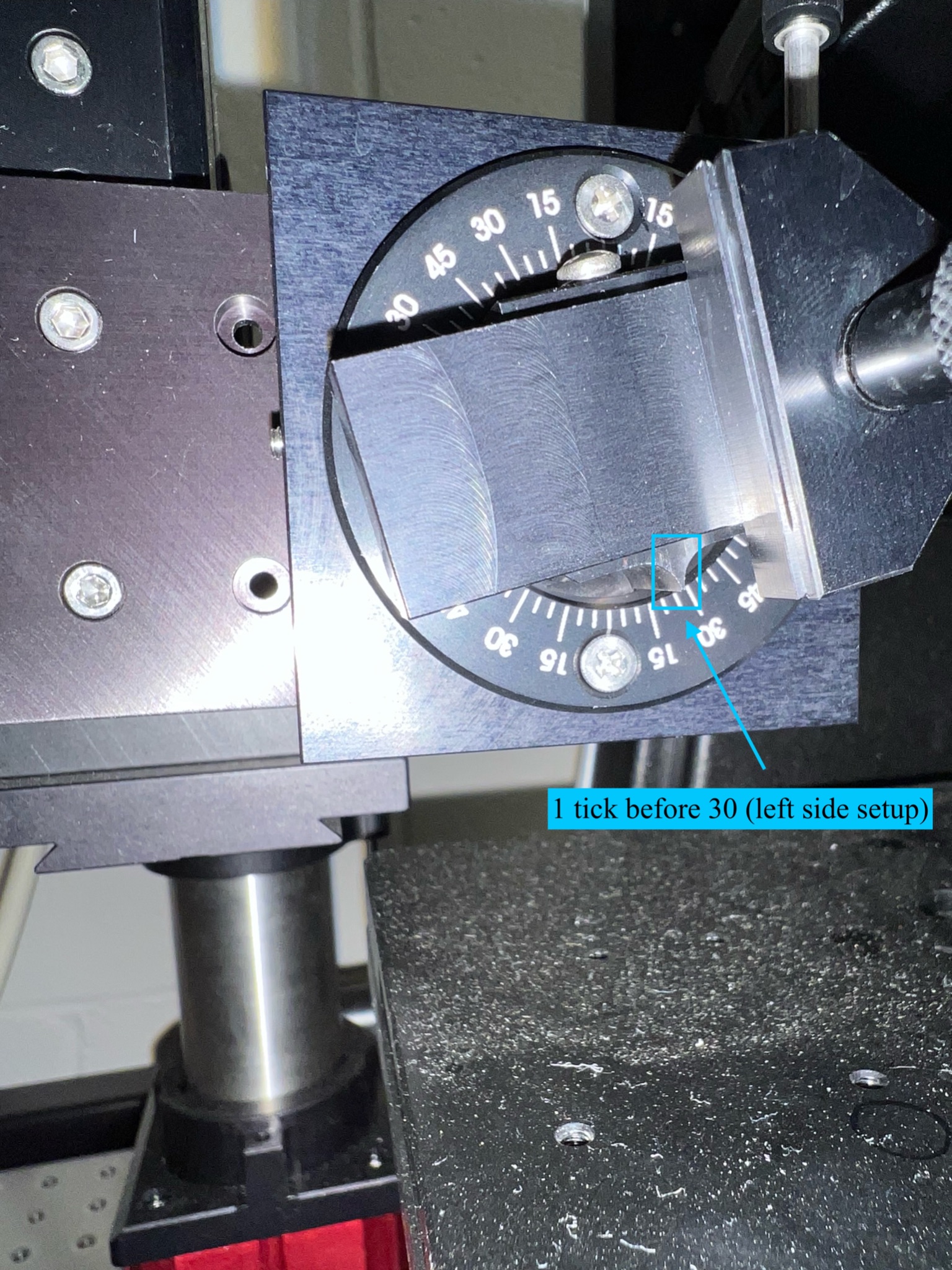


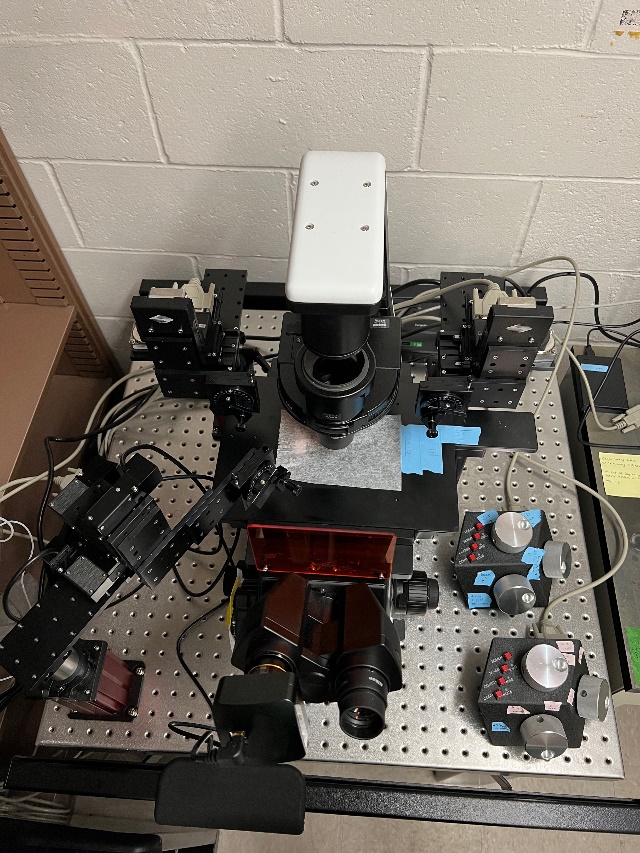

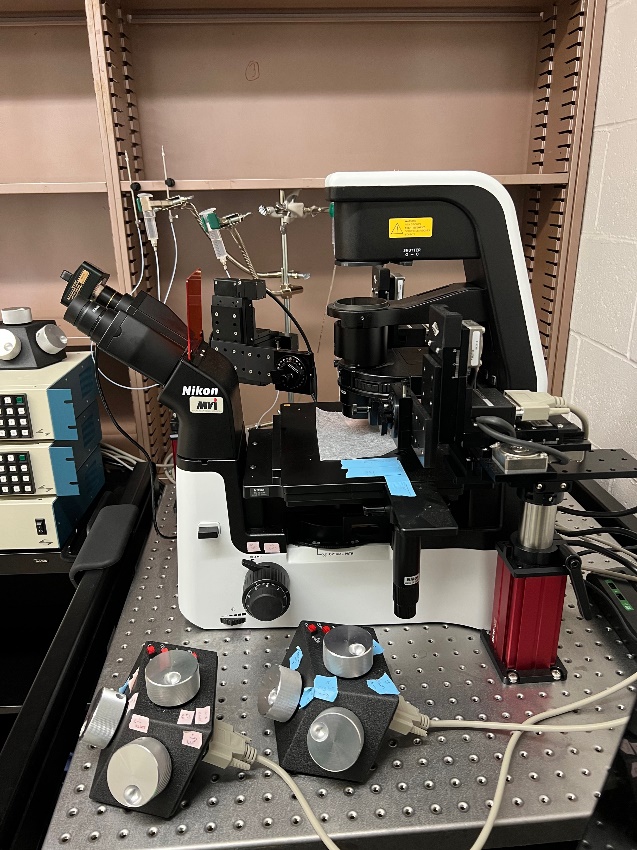


# Gravity well


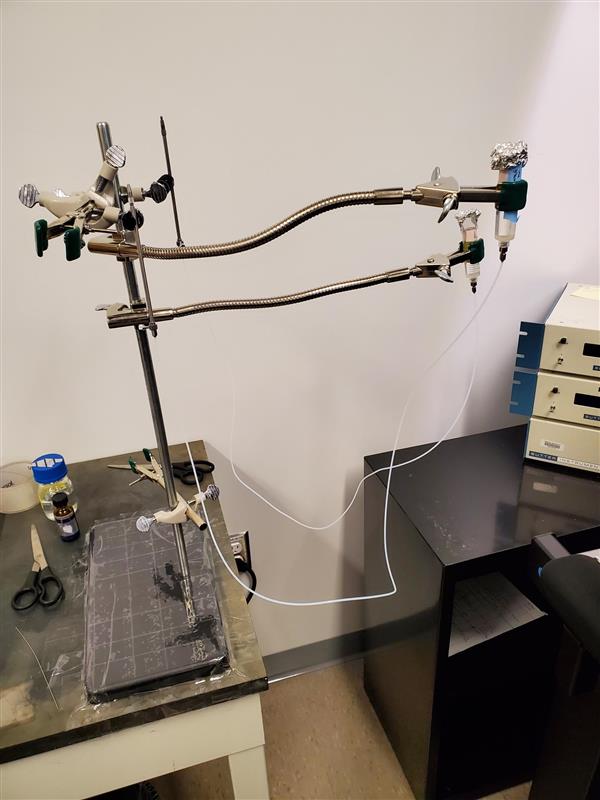
Our micromanipulation system uses a gravity well system to control the general flow direction and rate through the micropipette. This is a budget alternative to picopumps which can cost $1,000-$10,000 and can finely control suction and expel forces. Our micromanipulation system does not measure or report flow pressures or rates. Instead, measurements of force are based on pipette movements, deflections, and extensions applied to objects of interest, such as the nucleus.

- 1. Tubing ([either CT-1 or CT-2 tubing](http://products.narishige-group.com/group1/CT-1/injection/english.html))
  2. Tube connector ([CT-1 tube connector](http://products.narishige-group.com/group1/CI-1/injection/english.html))
  3. 10 mL Syringe with Luer lock ([89215-230](https://us.vwr.com/store/product/16167641/henke-ject-3-part-disposable-syringes-air-tite))
  4. Metal chem stand
  5. Flexible arm x2
  6. Large tubing = more flow through the system, less pressure. Requires bigger Δheight to change from pushing and sucking. Larger tubing has an inner diameter of 1/16" which is equal to about 1.5875mm.
  7. Smaller tubing = less flow through the system, higher pressure. Requires smaller Δheight in order to change from pushing and sucking. Smaller tubing item is from Narishige, item [CT-1](http://products.narishige-group.com/group1/CT-1/injection/english.html) with an inner diameter of 0.9mm. It is part of the [IM-H1 Injection](http://products.narishige-group.com/group1/IM-H1/injection/english.html) holder set.
     - The tubing that goes from the micropipette holder to the syringe was packaged in a circle, which we felt might create some problems with the ability for water to flow through properly. To fix this, I taped the tubing onto the wall with the micropipette holder on the lower end to allow gravity to stretch the tubing out to its full length, then applied tape to hold the tubing and micropipette holder to the wall. I left it overnight to allow the tubing to straighten out completely.
  8. Set up - This set up utilized two different Y axis adjustments. The first is that the metal arms that hold the syringes themselves were flexible and could be bent so that the syringes were higher or lower. Additionally the metal arms in how they were connected to the chem stand could be adjusted.
     - The best utilization of this setup involves using the chemstand to arm as the coarse adjustment, and the flexibility of the arms for smaller adjustments / to move the syringes out of the way when we need access to the area.

# Cell preparation

Cells must be prepared in conditions where a micropipette can access the cells.

- Culture cells in low profile cell culture dish that allows micropipette access ([FD3510](https://www.wpiinc.com/fd3510-100-fluorodish-cell-culture-dish-35mm-10mm-well-pkg-of-100))
  - - Alternatively, you can hand make a well with two O-rings (diameter 28 mm) paraffin wax coated and attached to a 1.5 coverslip glass.
- When cells are passaged use a technique to add cells as a dot off center to provide a differing confluence to choose an area with 60% confluency. Having too many cells will clog the micropipettes and ruin the experiment.
- As you cannot have contact between the pipette and the edges of the dish, consider that you want your areas of optimal confluency to be in the center of the dish to minimize accidental contact that breaks the pipette tips.
- For MEF V-/- cells no pre-treatments are necessary for nucleus isolation. For all other cell lines treat with treated with 1 µg/ml latrunculin A for 45 minutes before isolation to depolymerize actin to allow for nucleus isolation.

# Single nucleus isolation

Isolation of a nucleus form a live cells requires using micromanipulator controls, live phase imaging, and changes in gravity wells to control flow out or into the micropipette.

- - - Before adding dish of cells to microscope add oil to the 60X oil objective, add the dish or well containing cells, and touch the oil to the coverglass before moving to the 10X objective. Remember to lower the objective before switching.
    - To prepare for isolation, pull a spray pipette, cut to 4µm to 5µm in opening size and fill with 0.05 Triton percent by volume (see sections above for detilas).
    - The filled pipette is then loaded into the pipette holder. When loading into the pipette holder, you must make sure that the syringe is raised up above the stage so that the flow is down and water is dripping out of the pipette holder when you insert the pipette into the holder. It can be helpful to have a pair of flat tweezers with parafilm wrapped around the tips so that you can grab the pipette and use that to help insert the pipette into the pipette holder.
    - Before you snap the pipette holder into place on the MM set up, you should raise the MM all the way up so that the pipette doesn't make contact with the plate prematurely.
    - To find the pipette, start in 10x. Watch the pipette tip as you lower the MM (and therefore the pipette) into the media of the dish. Since you are using transmitted / diascopic light via phase, you can train your eye (outside of the microscope oculars) to know where the tip of the pipette is in relation to the circle of light on the imaging dish put out by the transmitted light bulb shining down. Once you find the pipette in 10x, confirm that you are touching the bottom of the dish (lower until you see the pipette slide), raise the pipette up a little bit so it doesn't get clogged with cells from the bottom of the plate, and then switch the objective to 60x. Stop and change your movement speed to fine and recenter the pipettes in the new FOV as necessary. Once the pipette is found in 60x, you can start to isolate a nucleus.
    - To pick a nucleus to isolate, you should consider the ease at which the nucleus will become free. Nuclei with visible filaments stretching over them or attached to the cell body can often be difficult as the filaments do not disintegrate with Triton spray. Visually, you should look for a nucleus that is of good size (about 15µm in length), and that is surrounded by cell body that has dark spots. The dark spots can act as an indicator that the Triton is working. To start spraying, lower the tip of the pipette until it is making contact with the cell body (not the periphery). Since the syringe is above the plate, Triton should spray out. This will be reflected in watching the dark spots of the cell body start to move and float away. When you start to isolate the nucleus, sometimes the cell body will collapse onto the nucleus making it impossible to fully isolate. To combat this, I use a method where I start spraying on one side of the nucleus, pick up the pipette, move to another side of the nucleus and spray there. By being consistent with the spray around the nucleus (as opposed to spraying and isolating 1 side completely and then beginning on the other side) the periphery of the nucleus remains free, which is important for grabbing onto the nucleus in more advanced MM techniques.
    - Sometimes, the spray pipette will have issues with the initial expulsion of triton. You can raise the pipette off the bottom of the glass, and use a rubber ball on the top of the syringe to create a rush of pressure that will clear any blockages and create flow in the downward direction. However, be aware that when you take the ball off of the syringe, you should avoid creating negative pressure that will cause the pipette to aspirate up gunk or cellular material from the dish causing a clog. Once you feel that you have established flow, you can bring the pipette back down to make contact with the cell body and start isolating.
    - When you are isolating a nucleus with the intent of using it for MM purposes, the nucleus does not need to be 100% isolated (floating away). Instead, you can isolate with 1 pipette, and have another pipette filled with PBS ready to grab onto the nucleus once you have chemically isolated the majority of the nucleus. Then, as long as you have the side closest to the PBS pull pipette free of any filaments or cell body, you can grab onto the nucleus (get on the same plane as the nucleus, go near the nucleus with the tip of the pipette, then lower the syringe so that the pipette begins to suck up. Once you see that the nucleus has been grabbed, raise the pipette up so that the nucleus gets lifted off the glass. Once the pipette and nucleus are out of focus (off the bottom), change the focal plane of the microscope. Check to see if removing the nucleus off the glass completed the isolation process. To do this, you can move the pipette and nucleus side to side, and see if any filaments are still attached to the nucleus and restricting its ability to move. If the nucleus is fully isolated, you can swap out the spray pipette and replace it with a pre-calibrated FP or whatever else you need to move forward with the experiment.

***For an actual nucleus isolation example, please refer to the main manuscript Figure 2.

# Single nucleus force measurement

Isolated single nuclei are ready for force measurements by following the steps below. We provide suggestions for imaging, tracking, analysis, and data handling along with an example of how to calculate force measurements from changes in force and pull pipette position.

- Attach the force pipette to the opposite side of the nucleus to the already attached pull pipette by dropping the corresponding gravity well. Be careful not to suck in too much and allow the nucleus to non-specifically adhere to the pipette walls.
- Keep the force pipette still and move the pull pipette to position the nucleus perpendicular to the pipettes preparing it for force-extension measurements. Make sure to return the force pipette to its original position by moving the pull pipette to relieve any extension or compression forces during this time. A tracking program during this step helps.
- You are now prepared for a micromanipulation force-extension measurement
- To track pipette position movement, take a movie or launch a live tracking program to track micropipette position. Suggestions below:
  - Imaging – AmScope base software, save as .bmp, .tiff, or .avi
  - Imaging – [µManager](https://micro-manager.org/) is a free to use software
  - Tracking/analysis – [FIJI (ImageJ)](https://imagej.net/software/fiji/) is a free use microscope analysis software
    - Line scans can be used to measure change in position between large time intervals
    - Kymographs can be used to see how position changes with time
    - It is possible that there is a free pipette tracking plug-in for µManager/FIJI
  - Data analysis/graphing – Microsoft Excel free use online
- Using a software program that talks to the Sutter Instrument MP-285, the MP-285’s own programing, or manual movements with the micromanipulator controller – move the pull pipette ~5 µm (short regime) or 10 µm or more (short and long regimes) and return the pull pipette to its original position. Record or live track during this movement.
- Run another force-extension measure when ready. Measurements are performed between 3-6 times on the same nucleus to provide an average nuclear spring constant.
- Using a tracking program, track the x position of both the force and pull pipettes during the experiment. Use the provided equations to provide force measurements. Example below:

| force pipette x position | pull pipette  x position | force pp  Δx | pull pp  Δx | Force = (Δx * k_fp_), k_fp_ = 1.5 nN/um | Extension =  (Δx pull – force) | Nuclear spring constant |
| --- | --- | --- | --- | --- | --- | --- |
| **µm** | **µm** | **µm** | **µm** | **nN** | **µm** | **nN/µm** |
| 12.0 | 23.5 | 0 | 0 | 0 | 0 |  |
| 12.1 | 23.9 | .1 | .4 | .15 | .3 | .5 |
| 12.2 | 24.3 | .2 | .8 | .3 | .6 | .5 |
| 12.3 | 24.7 | .3 | 1.2 | .45 | .9 | .5 |
| 12.4 | 25.1 | .4 | 1.6 | .6 | 1.2 | .5 |
| 12.5 | 25.5 | .5 | 2.0 | .75 | 1.5 | .5 |
| 12.6 | 25.9 | .6 | 2.4 | .9 | 1.8 | .5 |

***An actual experiment showing pipette movements during a single nuclear micromanipulation force-extension experiment, please refer to the main manuscript Figure 3.

# Other micromanipulation approaches

Micromanipulation apparatus can be used for more than nuclear spring constant measurements. Below we provide more details on some of these approaches.

- 1. Compression
     - The compression experiments were done with the nucleus remaining inside the cell body. This technique allowed us to apply compression to the nucleus using a micropipette, which we hypothesize would mimic force of actin compression and contraction. Using fluorescence allowed us to see the morphology of the nucleus because the cells had NLS-GFP (expressed endogenously as well as treated for cells that did not have this). The cell biology side of the lab was doing experiments to watch loss of signal occurring naturally via blebbing and rupture of nuclei, this MM setup allowed us to put pressure on the nucleus so that we could better understand rupture dynamics.
     - In order to do this, a spray pipette( #27) was pulled, cut, and loaded into the MM system in a similar way to the isolation technique described above. The pipette was first found in diascopic/trans light in 10x and then 60x. After confirming the location of the pipette, we switched into fluorescence. From here, we could find nuclei of different morphology. For each type of morphology and cell type, we would start by lowering the pipette onto the nucleus, then raising it back up. This was repeated multiple times. Then, for nuclei with abnormal morphology (blebs or otherwise), we would put the pipette down on the nucleus and then move the pipette through the nucleus, pushing nuclear material around. This allowed us to push volume into the bleb (areas of higher curvature) which would sometimes cause rupture or secondary blebs to form.
  2. Isolation shape/persistence
     - This technique was created to observe if morphological abnormalities, like blebs, were persistent after the nucleus was isolated out of the nucleus. This allowed us to see if deformations were plastic, or if by removing the nucleus from the bleb (and therefore removing the actin compression) the bleb would be reabsorbed into the cell body.
     - In order to observe change in morphology, pipettes were loaded with triton, and then found in 60x. The pipette was then lifted up so that it was out of focus and not spraying directly onto cells. The gravity well syringe was brought to a neutral height so that the pipette was not sucking in (getting clogged) but also wasn't spraying triton prematurely Cells of interest were brought into focus at 60x. Video acquisition began using a program connected to the microscope eyepiece camera (Our setup used AmScope). It is important to keep the perimeter of the nucleus in focus for measurement and shape tracking purposes. We isolated the nucleus using technique described in the isolation section. The video acquisition continued throughout the isolation process so that measurements could be taken throughout the process. Once the nucleus was fully isolated, the pipette was raised up and away from the nucleus so that the nucleus was not being sprayed onto with Triton. We chose to observe the nucleus over a set amount of time to see morphology change over time.
