# Supplementary material for: A Versatile Micromanipulation Apparatus for Biophysical Assays of the Cell Nucleus
Source: Cell Mol Bioeng. 2022 Sep 6;15(4):303–12. doi: 10.1007/s12195-022-00734-y (PMC9474788; doi:10.1007/s12195-022-00734-y)
Supplement: Supplementary file 9 — Supplementary file9 (DOCX 16 kb) [file 12195_2022_734_MOESM9_ESM.docx]

### Supplemental table 1. Data of nuclear spring constants.

Raw data of the chromatin short regime, chromatin + lamin A long regime, and strain stiffening (long/short) for all cell types listed in Figure 3.

### Supplemental protocols. Micromanipulation protocols.

Detailed point-by-point protocols and images for pulling, cutting, filling, micromanipulators, gravity well, cell preparation, single nucleus isolation, single nucleus force measurement, and other micromanipulation approaches.

### Supplemental movie 1. Micropipette pulling.

A Sutter Instruments Flaming/Brown micropipette puller P-97 is used to taper capillaries to produce micropipettes for use in micromanipulation experiments.

### Supplemental movie 2. Micropipette cutting via microforge and independent micropipette holder.

Micropipettes require cutting to make an open pipette tip with a defined size. We modified the approach for cutting micropipettes using the MF-200 WPI microforge and a custom pipette holder/positioner using Thorlabs parts.

### Supplemental movie 3. Front and back filling of a micropipette.

The micropipette must first be front filled using suction due to the narrow (micrometer) opening. Using a syringe and needle the rest of the micropipette is back filled. We use 0.05% Triton X-100 in PBS for isolation "Spray" micropipettes and just PBS for all other micropipettes.

### Supplemental movie 4. Loading of the micropipette into a micropipette holder and micromanipulator.

After pulling, cutting, and filling the micropipette is loaded into a holder and attached to the micromanipulator.

### Supplemental movie 5. Finding the micropipette in the microscope on 10X and 60X using phase microscopy.

Loaded micropipettes need to be positioned in microscope relative to objectives field of view so that they can be positioned for isolation, force measurement, and all other micromanipulation-based techniques.
